# Haemodynamic changes during radical nephrectomy with inferior vena cava thrombectomy: A pilot study

**DOI:** 10.1002/bco2.154

**Published:** 2022-04-28

**Authors:** Harshit Garg, Dharam Kaushik, Dawn Hui, Zachary Kahlenberg, Emily Vail, Lalithapriya Jayakumar, Hanzhang Wang, Deepak Pruthi, Ahmed M. Mansour, Michael Little

**Affiliations:** ^1^ Department of Urology University of Texas Health San Antonio San Antonio Texas USA; ^2^ Department of Cardiothoracic Surgery University of Texas Health San Antonio San Antonio Texas USA; ^3^ Department of Surgical Disciplines Baylor College of Medicine Houston Texas USA; ^4^ Department of Anesthesiology and Critical Care, Perelman School of Medicine University of Pennsylvania Philadelphia Pennsylvania USA; ^5^ Department of Vascular Surgery Vascular Health Partners Community Care Physicians Latham New York USA; ^6^ Cardiothoracic and Transplant Anesthesiology University of Texas Health San Antonio San Antonio Texas USA

**Keywords:** hemodynamic variation, inferior vena cava thrombectomy, radical nephrectomy, renal cell carcinoma, stroke volume variation

Renal cell carcinoma (RCC) is amongst the top 10 cancers in the United States, and approximately 10%–15% of these cancer cases are complicated by renal vein and inferior vena cava (IVC) thrombus.^1^ An aggressive surgical approach, that is, radical nephrectomy (RN) with IVC thrombectomy, has been associated with a 5‐year survival rate of 40%–68%.^2^ The venous tumour extension can directly occlude the IVC (entirely or partially), generate venous stasis to create bland thrombus or invade the IVC wall. Over time, progressive occlusion of the IVC leads to reliance on collateral blood flood flow through the lumbar and azygos veins. Intraoperatively, to achieve full control of the IVC for tumour thrombectomy, the surgeon must ligate and control both collateral vessels and occluding tributaries of the IVC, including lumbar, renal, accessory hepatic, retro‐hepatic and gonadal veins. In some instances, cardiopulmonary bypass (CPB) or deep hypothermic circulatory arrest is required.^2^ These cases are associated with significant hypotension from a combination of haemorrhage, venous occlusion and embolic events. While hypotension caused by haemorrhage and embolism may be easily detected by the surgical team or by intraoperative transesophageal echocardiography (TEE), the degree of hypotension associated with a decrease in preload due to sequential occlusion of the IVC and venous collaterals is not well characterized. The purpose of this study is to assess the haemodynamic changes that occur during operative steps of RN with IVC thrombectomy to aid the anesthesiologist in the management of expected intraoperative events.

We conducted a prospective observational pilot study of adult patients with RCC and tumour thrombus, who underwent RN with IVC thrombectomy, at a single academic tertiary care hospital in the United States. A sample size of 10 patients was selected a priori. The study was approved by the Institutional Review Board (reference number: HSC20190259E).

Comprehensive perioperative surgical care of these patients at our institution has been previously described.^3^ All procedures were performed by a single urologist with the assistance of cardio‐vascular surgeons, as indicated. The anaesthesia care included general endotracheal anaesthesia, arterial and central venous catheterization and noninvasive haemodynamic monitoring by an Edwards FloTrack® system arterial pulse contour analysis monitor. Crystalloids, blood products and vasopressor drugs were administered at the discretion of the attending anesthesiologists and were not standardized. TEE was used in all cases to determine the degree of IVC thrombus extension, to guide application of IVC clamps and to monitor for embolic events. Once TEE was placed, the cardiac anesthesiologist, urologist and vascular surgeon reviewed the extent of thrombus prior to making any incision. We also evaluated for IVC wall invasion, bland thrombus and distance of proximal limit of thrombus from hepatic veins. These small preoperative maneuvers and closed‐ loop communication assisted in determining potential problems during the surgery.

Patients' clinical, demographic and perioperative details were recorded. The Mayo clinic thrombus classification was used for describing the level of IVC thrombus. Intraoperative haemodynamic variables included mean arterial pressure (MAP) and stroke volume variation (SVV) measured from radial arterial lines, and cardiac index (CI) and systemic vascular resistance (SVR), as estimated from the FloTrak® device. Each haemodynamic outcome variable was measured at 10 different pre‐defined perioperative time points (Table 1). In patients who required intraoperative CPB, haemodynamic variables collected while on bypass were excluded.

**TABLE 1:** Clinical, demographic and perioperative details of patients, including variations in haemodynamic parameters at various surgical steps

Clinico‐demographic profile				
Age, years, median (IQR)	64 (59–70)			
Gender, (M/F), *n* (%)	7 (70%)/3 (30%)			
Body mass index, median (IQR), kg/m^2^	28.3 (26.6,34.3)			
Comorbidities				
Hypertension, *n* (%)	8 (80%)			
Diabetes Mellitus, *n* (%)	6 (60%)			
Stage of RCC (as per 8^th^ AJCC)				
Stage III, *n* (%)	8 (80%)			
Stage IV, *n* (%)	2 (20%)			
Level of tumour thrombus				
Level I, *n* (%)	1 (10%)			
Level II, *n* (%)	6 (60%)			
Level III, *n* (%)	1 (10%)			
Level IV, *n* (%)	2 (20%)			
Preoperative LVEF (%), median (IQR)	62.5 (60,67.5)			
Hospital stay, days, median (IQR)	8.5 (6–10)			

Intraoperative characteristics				
Operative time, minutes, median (IQR)	309 (189,480)			
Use of Cardiopulmonary Bypass, *n* (%)	2 (20%)			
Estimated blood loss, median (IQR)	1150 (400,5000)			
pRBC transfusion (units), median (IQR)	3 (1–6)			
Crystalloid (ml), median (IQR)	2000 (1500,3500)			
Norepinephrine equivalents^a^ received in OR, median (IQR)	587.5 (160,1400)			
Postoperative mechanical ventilation, *n* (%)	4 (40%)			

Intraoperative variation in haemodynamic parameters				
Surgical step	Mean arterial pressure (MAP), mm Hg	Cardiac index (CI), L/min/m^2^	Stroke volume variation (SVV), %	Systematic Vascular Resistance (SVR), mmHg*min/mL
1.Holding area/Prepping (pre‐operative)	98 (93,106)	—	—	—
2.Endotracheal intubation	78 (58,92)		—	—
3.Exposure of IVC (Baseline)	93 (80,101)	3.4 (3,4)	6 (5,8)	1058 (618–1333)
4.Renal artery ligation	83 (77.96)	2.9 (2.5,3.2)	9.5 (7,10)*	1123 (739–1316)
5.Ligation of lumbar vessels	75 (70,80)*	2.6 (2.1,3.0)*	15 (13.5,21)*	1116.5 (792–1450)
6. Clamping of contralateral renal vein	74 (69,87)	2.2 (2.1,2.7)	22 (15,26.5)	1207 (1153–1336.5)
7. Clamping IVC ± accessory hepatic veins	72 (65,87)*	2.2 (2,2.4)*	21 (19,23)*	1556 (1137–1625)*
8. Extraction of tumour thrombus	66 (54,72)	2.6 (2.2,2.9)	14 (8,24)	1094 (497–1671)
9. Closure of IVC and IVC reperfusion	89 (85,92)	3.1 (2.6,3.3)	11 (8,15)*	1047 (964–1304)
10.Abdominal skin closure	73 (71,76)*	2.5 (2.3,3)*	13 (10,17)*	978 (815–1162)

*Note*: AJCC—American Joint Committee on Cancer; CPB—cardiopulmonary bypass; EBL—estimated blood loss; ICU—intensive care unit; IVC—inferior vena cava; IQR—interquartile range; LVEF—left ventricular ejection fraction; OR—operating room, pRBC—packed red blood cell; RCC—renal cell carcinoma; SD—standard deviation.

^a^ 1ugnorepinephrine = 10 μg phenylephrine = 0.4 U Vasopressin.

* *p*value < 0.05 is considered as statistically significant (calculated using Wilcoxan rank‐sum test).

The mean (±standard deviation) or median (interquartile range), according to the normality of distribution, was reported for continuous variables. Each haemodynamic outcome at various surgical step was compared to baseline values (defined as the time of IVC exposure) using Wilcoxon rank‐sum tests. Analyses were conducted using Stata 13 and a *p* value of less than 0.05 was considered significant.

Ten patients undergoing open RN with IVC thrombectomy were included. Table 1: provides clinical, demographic and perioperative details along with variations in haemodynamic parameters. Cardiopulmonary bypass was utilized in two patients and those haemodynamic parameters while patients on bypass for these two patients were excluded. The individual haemodynamic parameters, blood loss, vasopressor used and volume of crystalloid and blood products used are shown in Table S1.

The haemodynamic parameters remained stable until ligation of lumbar vessels, when we observed a statistically significant increase in SVV (150% from baseline, *p* = 0.008) and a significant decrease in MAP (−19.4% from baseline, *p* = 0.012). The SVV remained significantly elevated till clamping of IVC (250% from baseline, *p* = 0.043) and then decreased by 47.6% at the IVC reperfusion (*p* = 0.027). The median CI decreased on ligation of lumbar vessels (−19.3%, *p* = 0.018) and clamping of IVC (−22.5%, *p* = 0.043). The SVR increased significantly from baseline at the time of clamping of hepatic veins and IVC (47.1%, p = 0.043). At the time of skin closure, the haemodynamic parameters were stable, but MAP and CI were significantly lower while SVV was higher as compared to baseline. In terms of perioperative outcomes, one patient suffered intraoperative pulmonary embolism, detected at the time of hepatic mobilization, and underwent pulmonary artery embolectomy after completion of oncological procedure. All patients received postoperative intensive care. No patients died during the index hospitalization.

Hence, we observed a progressive decrease in MAP and CI with an associated increase in SVV during surgical control and ligation of IVC branch vessels. These changes were most significant during ligation of lumbar vessels and clamping of the IVC.

The intraoperative monitoring and anaesthetic management during RN with IVC thrombectomy can be very challenging scenario. Therefore, it is important for the multidisciplinary team to be aware of haemodynamic changes occurring during caval manipulation. The major component of intraoperative monitoring includes fluid management, maintenance of stable haemodynamic parameters and immediate management of any possible embolism. The knowledge of the alterations in various haemodynamic parameters associated with each surgical step demand efficacious coordination and team effort. Goal‐directed fluid management during major abdominal surgeries has remained a key approach, and SVV evaluation has been proposed as a reliable predictor of fluid responsiveness to guide fluid therapy.^4^ The observed increase in SVV, which was most prominent during clamping of major abdominal veins and clamping of IVC, suggests a period of fluid responsiveness. This increase in SVV was also associated with a decrease in MAP and CI. In patients without significant haemorrhage, this period would also warrant the use of vasopressors, until the preload sequestered in the splanchnic and lower extremity vessels can be released, to prevent postoperative hypervolemia. The utility of SVV has been shown in major hepatic resections involving IVC and portal triad clamping.^5^

Fukazawa et al.^6^ advocated the application of cardiac anaesthesia principles, used in liver transplant settings, for the intraoperative management during RN and IVC thrombectomy. Morita et al.^7^ suggested an individual case‐by‐case basis flexible anaesthetic approach. In a review on perioperative management of RN with IVC thrombectomy, Woodruff et al.^8^ advocated a multi‐disciplinary approach but did not make any recommendations regarding intraoperative monitoring or anaesthetic management.

The literature on intraoperative haemodynamic variation in RN and IVC thrombectomy is limited. To the authors' best knowledge, this is the first study to systematically assess intraoperative haemodynamic alterations tied to pre‐defined surgical steps in a caval thrombectomy. In this study, we identified a progressive decrease in MAP and CI with an associated increase in SVV during surgical control and ligation of IVC branch vessels including lumbar vessels and clamping of IVC. The anticipation of these alterations not only prepares for the possible catastrophic outcomes but also allows pre‐emptive measures including volume replacement and initiation of vasopressor or inotropic drugs in a timely fashion. RN with IVC thrombectomy requires a cohesive approach by a multidisciplinary surgical team. Having an efficient communication system in real time between the surgical and anaesthesia team is critical for the success of this surgery. This pilot study made us aware of how much at each critical step of surgery‐communication matters. We have incorporated this model of close‐looped communication on all our thrombectomy cases.

However, this study has several limitations. It is a pilot observational study with limited sample size and without a comparator group. Importantly, anaesthetic management was not protocolized. The haemodynamic outcome variables may have been subject to systematic or random measurement error. Moreover, this study never aimed to identify a strategic approach or recommend an intraoperative anaesthetic management plan in patients undergoing RN with IVC thrombectomy. Finally, the clinical significance of statistically significant changes has not been established. Further studies may focus on correlating other outcome variables with these haemodynamic changes.

Despite these limitations, we believe that this is the first study to quantitatively describe haemodynamic changes during RN with IVC thrombectomy. These observations may not only inform anaesthetic care of future patients by helping anesthesiologists anticipate and treat changes in haemodynamics associated with planned surgical steps but may also improve multidisciplinary communication during surgery.

## AUTHORS' CONTRIBUTIONS

**Project development:** Dharam Kaushik and Michael Little; **data collection:** Harshit Garg and Zachary Kahlenburg; **data analysis:** Harshit Garg and Hanzhang Wang; **manuscript writing/editing:** Harshit Garg, Dharam Kaushik, Dawn Hui, Emily Vail, Lalithapriya Jayakumar, Deepak Pruthi, Ahmed M. Mansour and Michael Little.

## Supporting information


**Table S1:**Individual patient details of blood loss, vasopressor use, fluids used and haemodynamic parameters during radical nephrectomy with venous tumour thrombectomy
