# Supplementary material for: Haemodynamic changes during radical nephrectomy with inferior vena cava thrombectomy: A pilot study
Source: BJUI Compass. 2022 Apr 28;3(5):327–30. doi: 10.1002/bco2.154 (PMC9349591; doi:10.1002/bco2.154)
Supplement: Supplementary file 1 — Table S1:Individual patient details of blood loss, vasopressor use, fluids used and haemodynamic parameters during radical nephrectomy with venous tumour thrombectomy [file BCO2-3-327-s001.docx]

**Supplementary Table 1: Individual patient details of blood loss, vasopressor use, fluids used and hemodynamic parameters during radical nephrectomy with venous tumor thrombectomy**

| **Patient ID** | **Level of thrombus** | **Estimated Blood loss (EBL)/ml** | **Vasopressor used** | **Norepinephrine equivalents** received in OR** | **Crystalloid use, ml** | **pRBC transfusion, units** | **Hemodynamic**  **Parameter** | **Hemodynamic parameter at various steps of surgery** | | | | | | | | | | **Intraoperative event** |
| --- | --- | --- | --- | --- | --- | --- | --- | --- | --- | --- | --- | --- | --- | --- | --- | --- | --- | --- |
|  |  |  |  |  |  |  |  | Holding area/Prepping (pre-operative) | Endotracheal intubation | Exposure of IVC (Baseline) | Renal artery ligation | Ligation of lumbar vessels | Clamping of contralateral renal vein | Clamping IVC± accessory hepatic veins | Extraction of tumor thrombus | Closure of IVC and IVC reperfusion | Abdominal skin closure |  |
| **1** | III | 3000 | Norepinephrine | 565 | 2000 | 6 | **MAP** | 126 | 102 | 119 | 57 | 66 | 69 | 60 | 71 | 85 | 72 | Bleeding from lumbar vessel |
|  |  |  |  |  |  |  | **SVR** |  |  | 1016 | 803 | 784 | 1221 | 1137 | 1671 | 1042 | 922 |  |
|  |  |  |  |  |  |  | **SVV** |  |  | 6 | 18 | 19 | 21 | 21 | 19 | 8 | 10 |  |
|  |  |  |  |  |  |  | **CI** |  |  | 4 | 2.4 | 3 | 2.2 | 2.2 | 1.9 | 3.3 | 3 |  |
| **2** | II | 400 | Norepinephrine | 265 | 3500 | 2 | **MAP** |  | 44 | 80 | 77 | 74 | * | 56 | 47 | 64 | 72 | Uneventful |
|  |  |  |  |  |  |  | **SVR** |  |  | 510 | 574 | 507 | * | 608 | 497 | 554 | 449 |  |
|  |  |  |  |  |  |  | **SVV** |  |  | 5 | 9 | 13 | * | 19 | 29 | 15 | 14 |  |
|  |  |  |  |  |  |  | **CI** |  |  | 4.1 | 3.6 | 4 | * | 2.4 | 2.5 | 3.3 | 4.5 |  |
| **3** | II | 300 | Norepinephrine | 160 | 2000 | 1 | **MAP** | 93 | 75 | 76 | 74 | 70 | * | 65 | 61 | * | 71 | Uneventful |
|  |  |  |  |  |  |  | **SVR** |  |  | 856 | 803 | 813 | * | 821 | 861 | * |  |  |
|  |  |  |  |  |  |  | **SVV** |  |  | 5 | 10 | 12 | * | 15 | 9 | * | 12 |  |
|  |  |  |  |  |  |  | **CI** |  |  | 4.1 | 3 | 3.0 | * | 2.8 | 3.1 | * | 2.7 |  |
| **4**  **(on CPB)** | IV | 5000 | Norepinephrine + Vasopressin | 1400 | 1500 | 25 | **MAP** | 80 | 80 | 68 | 77 | 76 | Exc | Exc | Exc | Exc | 65 | Dense pericapusular adhesions. Bleeding in inter-aortocaval region and bear right hepatic vein |
|  |  |  |  |  |  |  | **SVR** |  |  | 582 | 1024 | 800 | Exc | Exc | Exc | Exc | 815 |  |
|  |  |  |  |  |  |  | **SVV** |  |  | 7.5 | 9 | 13.9 | Exc | Exc | Exc | Exc | 10.6 |  |
|  |  |  |  |  |  |  | **CI** |  |  | 4 | 3 | 3 | Exc | Exc | Exc | Exc | 3 |  |
| **5** | I | 200 | Norepinephrine | 21.75 | 1000 | 0 | **MAP** | 61 | 58 | 98 | 100 | 98 | 96 | 87 | 73 | 92 | 73 | Uneventful |
|  |  |  |  |  |  |  | **SVR** |  |  | 1333 | 1255 | 1200 | 1193 | * | 1094 | 1052 | 978 |  |
|  |  |  |  |  |  |  | **SVV** |  |  | 3 | 10 | 13 | 9 | * | 7 | 6 | 9 |  |
|  |  |  |  |  |  |  | **CI** |  |  | 3 | 3.2 | 3.2 | 3.1 | * | 2.7 | 2.8 | 2.5 |  |
| **6** | II | 1500 | Norepinephrine + Vasopressin | 1442.5 | 3500 | 4 | **MAP** | 106 | 92 | 80 | 88 | 78 | 78 | 76 | * | 89 | 88 | Pericapsular adhesions |
|  |  |  |  |  |  |  | **SVR** |  |  | 618 | 739 | 1114 | 1114 | 931 | * | 964 | 1013 |  |
|  |  |  |  |  |  |  | **SVV** |  |  | 12 | 13 | 23 | 23 | 23 | * | 24 | 15 |  |
|  |  |  |  |  |  |  | **CI** |  |  | 3.5 | 3.5 | 2.2 | 2.2 | 2.4 | * | 3.4 | 2.3 |  |
| **7** | II | 800 | Norepinephrine + Vasopressin | 662 | 3000 | 1 | **MAP** | 94 | 69 | 87 | 62 | 67 | 68 | 68 | * | * | 73 | Unevenftul |
|  |  |  |  |  |  |  | **SVR** |  |  | 1273 | 1326 | 1119 | 1452 | 1625 | * | * | 1518 |  |
|  |  |  |  |  |  |  | **SVV** |  |  | 6 | 7 | 14 | 30 | 14 | * | * | 24 |  |
|  |  |  |  |  |  |  | **CI** |  |  | 3 | 2.3 | 2.5 | 1.9 | 2.4 | * | * | 2.2 |  |
| **8**  **(on CPB)** | IV | 12000 | Norepinephrine + Vasopressin+ Phenylephrine | 609.75 | 5000 | 17 | **MAP** | 98 | 117 | 113 | 105 | 101 | Exc | Exc | Exc | Exc | 66 | Locally advanced tumor with extensive parasitic vessels, had splenic tear and bleeding |
|  |  |  |  |  |  |  | **SVR** |  |  | 1742 | 1358 | 1363 | Exc | Exc | Exc | Exc | 640 |  |
|  |  |  |  |  |  |  | **SVV** |  |  | 8 | 7 | 11 | Exc | Exc | Exc | Exc | 17 |  |
|  |  |  |  |  |  |  | **CI** |  |  | 2.2 | 2.6 | 2.6 | Exc | Exc | Exc | Exc | 2.5 |  |
| **9** | II | 10000 | Norepinephrine + Vasopressin+ Phenylephrine | 2827.5 | 3000 | 11 | **MAP** | 104 |  | 100 | 83 | 82 | * | 72 | * | 101 | 77 | Locally advanced tumor with intense desmoplastic reaction, extensive parasitic vessels and spleen plastered with tumor. Patient had intraoperative pulmonary embolism and underwent pulmonary artery embolectomy after completion of IVC thrombectomy |
|  |  |  |  |  |  |  | **SVR** |  |  | 1714 | 1316 | 1537 | * | 1756 | * | 1650 | 1463 |  |
|  |  |  |  |  |  |  | **SVV** |  |  | 10 | 10 | 16 | * | 23 | * | 13 | 19 |  |
|  |  |  |  |  |  |  | **CI** |  |  | 2.3 | 2.5 | 2.1 | * | 2 | * | 1.8 | 1.9 |  |
| **10** | II | 600 | Norepinephrine | 148 | 2000 | 0 | **MAP** | 112 | 92 | 101 | 96 | 72 | * | 81 | * | 89 | 76 | Uneventful |
|  |  |  |  |  |  |  | **SVR** |  |  | 1058 | 1222 | 1626 | * | 1556 | * | 1304 | 1162 |  |
|  |  |  |  |  |  |  | **SVV** |  |  | 3 | 3 | 27 | * | 19 | * | 9 | 9 |  |
|  |  |  |  |  |  |  | **CI** |  |  | 3.3 | 2.9 | 1.7 | * | 2 | * | 2.6 | 2.5 |  |

CI- Cardiac Index (L/min/m^2^), CPB- Cardiopulmonary Bypass, IVC- Inferior Vena cava, MAP- Mean Arterial Pressure(mm Hg), pRBC- packed Red Blood cells units, SVR- Systemic Vascular Resistance (mm Hg*min/L), SVV- Stroke Volume Variation(%)

Exc- Excluded readings as patient was on cardiopulmonary bypass, *- Missing data

**-1ugnorepinephrine= 10 ug phenylephrine= 0.4 U Vasopressin
